# Delayed presentation of traumatic cervical epidural hematoma: a case report and review of the pertinent literature

**DOI:** 10.1093/omcr/omae086

**Published:** 2024-08-06

**Authors:** Taiki Isaji, Tadato Yukiue, Takayuki Amano

**Affiliations:** Department of Neurosurgery, Nagoya Tokushukai General Hospital, 2-52 Kozojikita, Kasugai, Aichi 487-0016, Japan; Department of Neurosurgery, Nagoya Tokushukai General Hospital, 2-52 Kozojikita, Kasugai, Aichi 487-0016, Japan; Department of Neurosurgery, Nagoya Tokushukai General Hospital, 2-52 Kozojikita, Kasugai, Aichi 487-0016, Japan

**Keywords:** trauma, spinal epidural hematoma, delayed presentation

## Abstract

A delayed presentation of traumatic spinal epidural hematoma (SEH) is a rare disease in which most patients are asymptomatic for days to weeks after the injury, followed by pain and then a neurological deficit. A 66-year-old woman who suffered a fractured right clavicle due to a bicycle accident 42 days previously, presented with left shoulder pain and left hemiplegia. The manual muscle test (MMT) scores of the left upper and lower limbs were all 1. Computed tomography and magnetic resonance imaging showed no cervical fracture but showed cervical epidural hematoma. She underwent surgery for the removal of the SEH. Her MMT score improved to 4 at 10 days after surgery. Even in cases with a delayed presentation, suspecting SEH can help clinicians make an early diagnosis. Additionally, the prompt surgical evacuation of the SEH can lead to favorable neurological outcomes in symptomatic cases.

## Introduction

Spinal epidural hematoma (SEH) is a rare condition. Post-traumatic SEH accounts for less than 1%–1.7% of all spinal injuries [1–6]. In particular, delayed presentation due to traumatic SEH is even rarer, with most patients experiencing an asymptomatic period of days to weeks after the injury, followed by pain and then a neurological deficit. [7, 8].

Because traumatic SEH may progressively worsen, and the prognosis may be poor without surgical treatment, a rapid and accurate diagnosis is needed in cases of suspected SEH. An early diagnosis and prompt surgical evacuation of the SEH in symptomatic cases can lead to favorable neurological outcomes. We herein report a case of traumatic SEH with a delayed presentation 42 days after injury who demonstrated a good outcome.

## Case report

**Figure 1:**
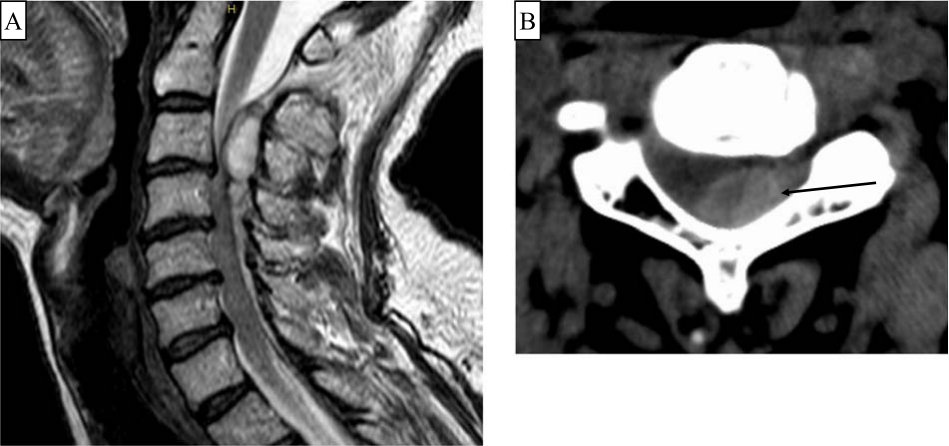
(**A**) T2-weighted Magnetic resonance imaging (MRI) showing an epidural hematoma at the C2/3 to C4 level. (**B**) Computed tomography (axial image) showing a hematoma predominantly on the left side at the C3 level (arrow).

**Figure 2:**
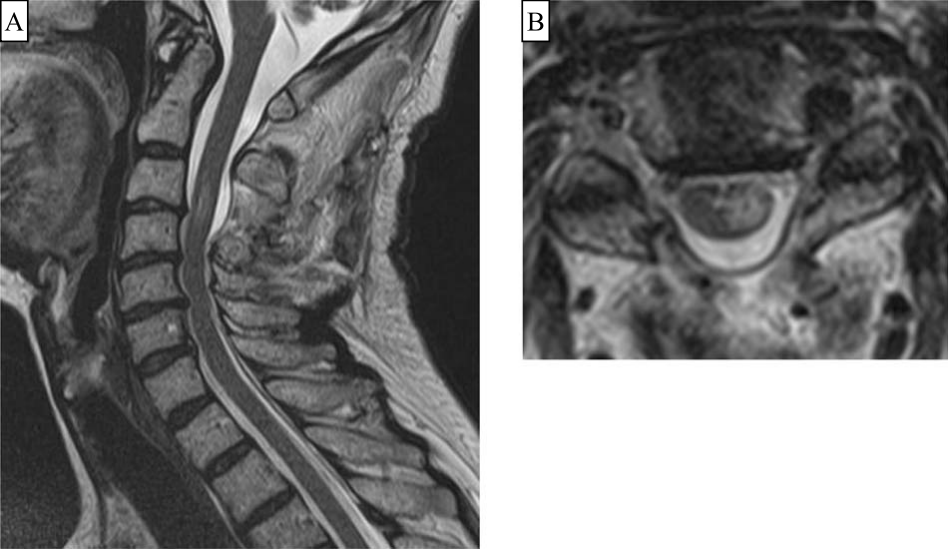
(**A** and **B**) Two days after surgery, T2-weighted Magnetic resonance imaging (sagittal and axial images) show decompression of the spinal cord and intramedullary high signal intensity in the left half at the C3 level.

A 66-year-old woman presented with left shoulder pain and left hemiplegia an hour later. The manual muscle test (MMT) scores of the left upper and lower limbs were all 1. Her deep tendon reflex (DTR) showed no hyperreflexia on the bilateral upper limbs, her right patella tendon reflex showed no hyporeflexia, and the left reflex was normal. Her trauma history was a fracture of the right clavicle due to a bicycle accident 42 days prior. The patient had no history of receiving antithrombotic therapy. Computed tomography (CT) and magnetic resonance imaging (MRI) showed no cervical fracture; however, an epidural hematoma dorsal to the spinal cord was observed at the level of C2/3 to C4 (Fig. 1). We performed C2 laminotomy, C3 laminectomy, and C4 hemilaminectomy to remove epidural hematoma. An epidural hematoma was observed between the yellow ligament and dura mater. Vascular malformations and tumor-like tissues were not observed under an operative microscope. Her postoperative MMT score improved to 3 on the day after surgery and to 4 10 days after surgery. Postoperative MRI revealed decompression of the spinal cord and intramedullary high signal intensity at the C3 level (Fig. 2). She was transferred to a rehabilitation hospital 13 days after surgery.

## Discussion

The incidence of spontaneous SEH is estimated to be 0.1 per 100 000 patients per year [7]. It has also reported that 40% of all SEHs have no definite etiological factors; these cases are considered to be “spontaneous” [4]. In the relevant English literature, there are only eight reported cases of delayed presentation of traumatic SEH (Table 1). Seven of these eight cases and the present patient presented with either pain at the SEH site or neuropathic pain. In addition, in all nine patients including the present case, motor weakness occurred either with pain simultaneously or subsequently. SEH should therefore be considered in the differential diagnosis even in cases with a delayed presentation and associated with pain and motor weakness after trauma.

**Table 1:** The characteristics of delayed presentation traumatic SEH

Age	Sex	Period from injury to onset	Location of SEH	Symptom	Fracture	Therapy	Reference
20	M	3 days	Dorsal to the spinal cord at C6-T2	Bilateral leg numbness and weakness, low grade fever and generalized malaise	None	Surgery	[1]
79	F	6 weeks	Dorsal to the spinal cord at T10-L2	Sever back pain, progressive motor weakness, and paresthesia of both lower limbs	T11 compression fracture	Surgery	[2]
22	M	10 days	Ventral to the spinal cord at C6-T6	Sudden onset of severe neck and upper back pain, loss of sensation and motor function in bilateral lower extremities	None	Conversation	[3]
77	F	16–17 days	Dorsal to the spinal cord at T11-T12	Severe back pain followed by bilteral lower extremity paraplegia and sensory loss	T10 compression fracture	Surgery	[4]
57	F	1 week	Dorsal to the spinal cord at C3-T2	Progressive interscapular pain and right-sided hemiparesis	None	Surgery	[5]
78	F	2 months	Dorsal to the spinal cord at T9-T12	Severe back pain, motor weakness as well as pain of bilateral lower limbs, and bladder-bowel dysfunction	L1 compression fracture	Surgery	[6]
12	M	3 weeks	Dorsal to the spinal cord at C7/T1-T4	Intence back pain, a loss of motor function in both legs as well as bilateral numbness and tingling in the lower extremities	None	Surgery	[7]
21	M	27 days	Ventral to the spinal cord at C7-T2	Severe pain in his right arm and both shoulders, and muscle weaknesss in the right fingers and bilateral legs	None	Surgery	[10]
66	F	42 days	Dorsal to the spinal cord at C2/3-C4	Left shoulder pain followed by left hemiplegia	None	Surgery	present case

SEH, spinal epidural hematoma; M, Male; F, Female; C, Cervical; T, Thoratic; L, Lumbar.

SEH is caused by vascular malformations, iatrogenic disorders, pregnancy, neoplasms, and trauma. In the spinal epidural space, the venous plexus is thin-walled, valveless, and fragile. Even minor trauma can cause a hematoma due to elevated pressure of the venous plexus, in addition to direct injury to the vessels. Hemorrhage from the injured venous plexus is less likely to cause spinal cord compression and tends to disappear spontaneously. Kerslake et al. reported that MRI performed within three weeks after spinal trauma demonstrated SEH in 17 of 44 patients. However, MRI performed more than three weeks after trauma did not show SEH in any patient. Therefore, it is suggested that traumatic SEH is resolved within three weeks after trauma in most cases [6, 9]. Although the exact mechanism by which a delayed spinal epidural hematoma develops remains controversial, it is considered to be due to the following two mechanisms. One is that hemolysis of the initial clot on the dura mater occurs a few weeks after the trauma and leads to arterial rebleeding into the extradural space [10]. The other is the migration of fluid, including hemorrhage from osteoporotic vertebral collapse to the epidural space during daily motion. Kummell’s disease is avascular necrosis of the vertebrae after spinal compression fractures. It can also lead to chronic SEH [2, 6]. In the relevant literature, three cases in which delayed neurological deficits due to SEH developed from compression fracture have been reported [2, 4, 6].

MR studies are very sensitive for identifying soft tissue injuries. Therefore, MRI is essential for the early diagnosis of SEH, which is treated by surgery and conservative management. Eight cases with the delayed presentation of traumatic SEH were surgically treated. Another patient underwent conservative management. Each treatment resulted in improvement for all eight patients.

For SEH cases with only mild symptoms and no progressive symptoms, conservative therapy should be considered first. Surgical treatment is recommended for patients with severe symptoms. In the present case, early surgical treatment resulted in good outcomes.

In conclusion, spine surgeons and emergency physicians should be aware of delayed traumatic SEH. MRI should be routinely performed when patients with a history of trauma have symptoms suggestive of traumatic SEH.
